# Zoonotic bacterial and parasitic intestinal pathogens in foxes, raccoons and other predators from eastern Germany

**DOI:** 10.1111/1758-2229.13261

**Published:** 2024-05-15

**Authors:** Sonja Kittl, Caroline F. Frey, Isabelle Brodard, Nadia Scalisi, Maria Elena Vargas Amado, Andreas Thomann, Peter Schierack, Joerg Jores

**Affiliations:** ^1^ Vetsuisse Faculty, Institute of Veterinary Bacteriology University of Bern Bern Switzerland; ^2^ Vetsuisse Faculty, Institute of Parasitology University of Bern Bern Switzerland; ^3^ Department of Geography University of Zürich Zürich Switzerland; ^4^ Swiss Federal Research Institute WSL Birmensdorf Switzerland; ^5^ Faculty Environment and Natural Sciences, Institute of Biotechnology Brandenburg University of Technology Cottbus‐Senftenberg Senftenberg Germany; ^6^ Faculty of Health Sciences Brandenburg Brandenburg University of Technology Cottbus‐Senftenberg Senftenberg Germany; ^7^ Multidisciplinary Center for Infectious Diseases University of Bern Bern Switzerland

## Abstract

In this study, we investigated faecal specimens from legally hunted and road‐killed red foxes, raccoons, raccoon dogs, badgers and martens in Germany for parasites and selected zoonotic bacteria. We found that *Baylisascaris procyonis*, a zoonotic parasite of raccoons, had spread to northeastern Germany, an area previously presumed to be free of this parasite. We detected various pathogenic bacterial species from the genera *Listeria*, *Clostridium* (including *baratii*), *Yersinia* and *Salmonella*, which were analysed using whole‐genome sequencing. One isolate of *Yersinia enterocolitica* contained a virulence plasmid. The *Salmonella* Cholerasuis isolate encoded an aminoglycoside resistance gene and a *parC* point mutation, conferring resistance to ciprofloxacin. We also found tetracycline resistance genes in *Paeniclostridium sordellii* and *Clostridium baratii*. Phylogenetic analyses revealed that the isolates were polyclonal, indicating the absence of specific wildlife‐adapted clones. Predators, which scavenge from various sources including human settlements, acquire and spread zoonotic pathogens. Therefore, their role should not be overlooked in the One Health context.

## Graphical abstract

Our study involved the examination of faecal samples from wild predators in eastern Germany to identify parasitic and bacterial pathogens. In our investigation, we found *Baylisascaris procyonis* in raccoons, a parasite known to cause a potentially lethal disease in humans. Additionally, we discovered a potential reservoir for *Clostridium baratii* in these wild predators. Toxigenic strains of this bacterium can lead to botulism in humans. In terms of antimicrobial resistance genes, our observations revealed a limited range and low prevalence in the bacterial genera *Yersinia*, *Listeria*, *Clostridium* and *Salmonella*.

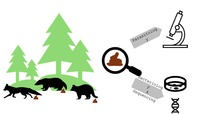

## INTRODUCTION

Predators such as foxes, raccoons and martens, although being wild, approach urban settlements on a regular basis, where they can pick up or shed zoonotic pathogens. The raccoon roundworm *Baylisascaris procyonis* is such a pathogen, which has been reported from raccoons in southern and central Germany, but not in the state of Brandenburg in eastern Germany (Heddergott et al., 2020). It is usually benign in raccoons but can cause neural larva migrans in paratenic hosts such as humans. The raccoon is not native to Germany and since its release into the wild in the last century, its population and territorial coverage have increased massively. Therefore, it is safe to assume that parasites and microorganisms associated with it spread over time as well.

While *Clostridium perfringens* is a normal inhabitant of the gut even of wild and domestic carnivores (Silva & Lobato, 2015), knowledge about the carriage of other *Clostridium* species is rather patchy, if existent at all. Particularly for *Clostridium baratii*, which is a sporadic agent of infant botulism, environmental reservoirs or reservoir hosts have not been identified (Halpin et al., 2018; Silva‐Andrade et al., 2022). Generally, data on the carriage of different pathogens in wild animals are to a great extent lacking and such knowledge gaps need to be filled for a One Health approach to control infectious diseases. Other pathogens such as *Salmonella enterica* subsp. *enterica* (Uelze et al., 2021), *Yersinia enterocolitica* (Carella et al., 2022) and *Listeria* spp. (Nowakiewicz et al., 2016) have been reported from predators, but their genomic sequences are largely missing so far, making comparisons to human isolates difficult. This study aimed to investigate the carriage of parasites and selected bacterial pathogens, including *Clostridium* spp., *Yersinia* spp., *Listeria* spp. and *Salmonella* using faecal specimens of different predators legally hunted or road‐killed in Eastern Germany. These data will assist in estimating the risks of getting infected by selected zoonotic pathogens investigated here and spread by wild predators.

## EXPERIMENTAL PROCEDURES AND RESULTS

**FIGURE 1:**
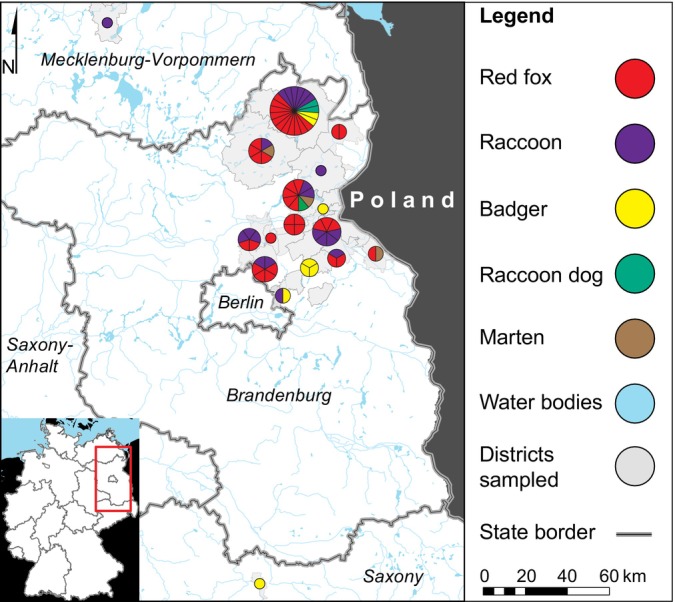
Map of the region under investigation. Number of tested animals are given next to the study sites. Map was created using Quantum GIS (http://qgis.osgeo.org) and data from http://www.naturalearthdata.com.

From 2021 to 2022, we sampled altogether 202 specimens from foxes, raccoons, badgers, raccoon dogs and martens legally hunted or road‐killed in Germany and investigated the latter for serological signatures and ongoing infections with *Leptospira* spp. (Kuhnert et al., 2024). As part of the sampling, we obtained 75 faecal specimens from a fraction of the animals, which were the subject of the current study (Figure 1). Specimens were tested for the presence of parasites and bacterial pathogens belonging to the genera *Yersinia*, *Salmonella*, *Listeria*, *Clostridium* and *Paeniclostridium*. The investigated animals included 39 red foxes (*Vulpes vulpes*), 22 raccoons (*Procyon lotor*), eight badgers (*Meles meles*), three raccoon dogs (*Nyctereutes procyonoides*) and three martens (*Martes martes*).

Faecal specimens were collected from the carcasses in the eastern German states of Brandenburg, Saxony and Mecklenburg‐Vorpommern (Figure 1), and stored in 1.5 mL reaction tubes at −20°C until further use. Parasites were diagnosed by the combined sedimentation‐flotation method (Deplazes et al., 2016). Bacteria were isolated in an accredited veterinary diagnostic laboratory using standard methods. Briefly, *Salmonella* were enriched in Mkttn Broth + Novobiocin (Oxoid) media and *Yersinia* as well as *Listeria* were cold‐enriched prior to culture on Yersinia Selective Medium (CIN) (Oxoid) for 96 h at 30°C and Palcam Medium (Oxoid) for 48 h at 37°C, respectively. Clostridia were isolated by anaerobic culture on Membrane Clostridium Perfringens Agar (Oxoid) and Brucella Blood Agar with Hemin and Vitamin K1 (BD) for 24 and 48 h, respectively. Preliminary species designation was done for Listeria via Vitek 2 (Biomerieux) and for the other bacteria by MALDI‐TOF MS (Bruker). Genomic DNA was extracted from cultures using the QIAamp DNA Mini Kit (Qiagen) (*Clostridia*) or according to Pitcher et al. (1989). *C. perfringens* pathotype typing was done using quantitative real‐time PCR according to Albini et al. with modifications (Albini et al., 2008). Genomes of all bacteria reported in this study, except *C. perfingens*, were sequenced using PacBio and assembled at the Lausanne Genomic Technologies Facility as described recently (Akarsu et al., 2022). Complete genome sequences were submitted to GenBank (PRJNA961371). Assemblies were screened for virulence and resistance genes using ABRicate 0.8 with the ResFinder database. Core genome SNP trees were generated using snippy 4.6.0 and visualised in MEGA 11.0.13. Pan‐genome analyses were performed with roary 3.13.0 and R‐package pagoo (Ferres & Iraola, 2021; Sitto & Battistuzzi, 2020). Multi‐locus sequence typing (MLST) alleles were obtained from pubmlst.org.

A total of 3 out of 22 raccoon samples tested positive for *B. procyonis* showing a north‐eastern expansion of the parasite in Germany besides a spectrum of other parasites (Supporting Information S1).

**FIGURE 2:**
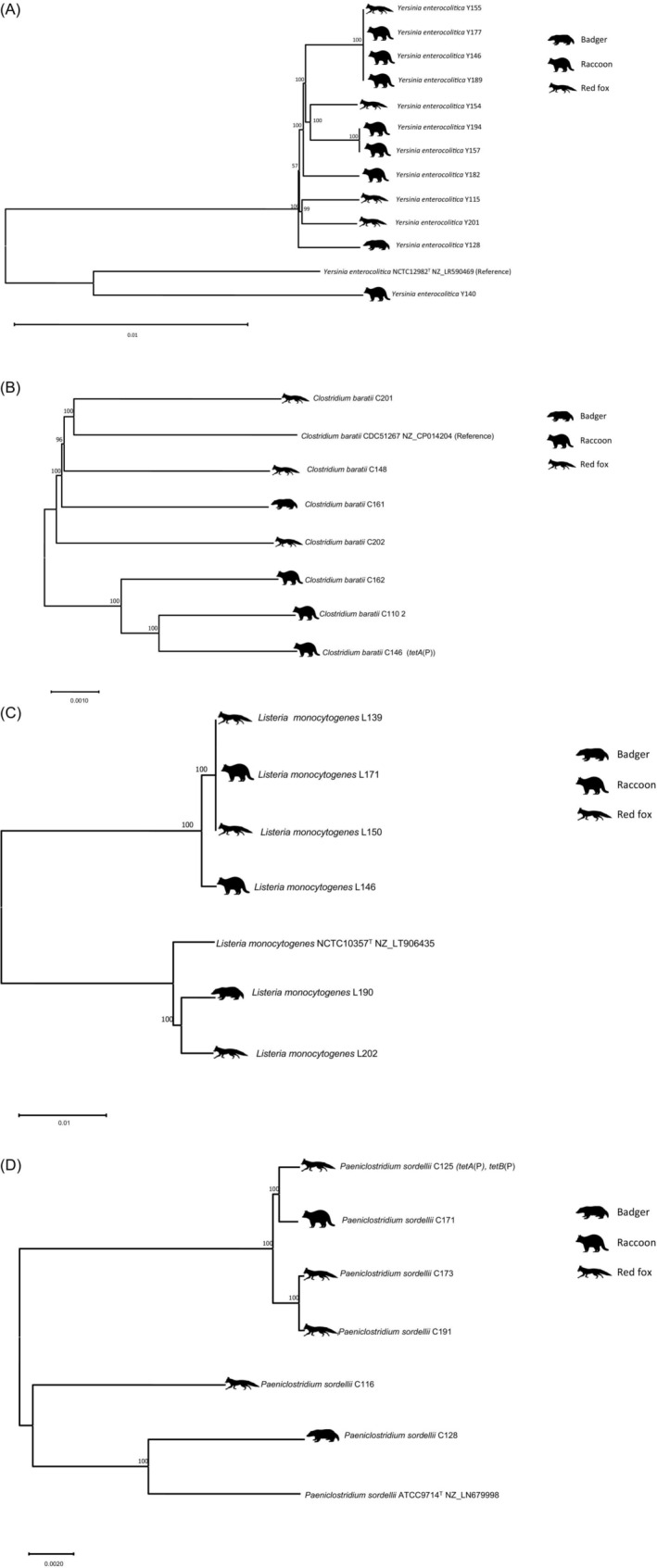
Phylogenies of bacterial pathogens isolated. (A) Phylogeny of *Yersinia enterocolitica* isolates. The host species is indicated with icons. The tree is based on core genome single nucleotide polymorphisms (SNPs) determined with snippy 4.6.0. Clustering was inferred with neighbour‐joining. The percentage of replicate trees in which the associated taxa clustered together in the bootstrap test (100 replicates) is shown above the branches. The evolutionary distances were computed using the Maximum Composite Likelihood method and are in the units of the number of base substitutions per site. The rate variation among sites was modelled with a gamma distribution (shape parameter = 1). There were a total of 3,375,159 positions in the final dataset. Evolutionary analyses were conducted in MEGA11. (B) Phylogeny of *Clostridium baratii* isolates. The host species is indicated with icons. The tree is based on core genome SNPs determined with snippy 4.6.0. Clustering was inferred with neighbour‐joining. The percentage of replicate trees in which the associated taxa clustered together in the bootstrap test (100 replicates) is shown above the branches. The tree is drawn to scale, with branch lengths in the same units as those of the evolutionary distances used to infer the phylogenetic tree. The evolutionary distances were computed using the Maximum Composite Likelihood method and are in the units of the number of base substitutions per site. The rate variation among sites was modelled with a gamma distribution (shape parameter = 1). There were a total of 2,643,672 positions in the final dataset. Evolutionary analyses were conducted in MEGA11. (C) Phylogeny of *Listeria monocytogenes* isolates. The host species is indicated with icons. The tree is based on core genome SNPs determined with snippy 4.6.0. Clustering was inferred with neighbour‐joining. The percentage of replicate trees in which the associated taxa clustered together in the bootstrap test (100 replicates) is shown above the branches. The tree is drawn to scale, with branch lengths in the same units as those of the evolutionary distances used to infer the phylogenetic tree. The evolutionary distances were computed using the Maximum Composite Likelihood method and are in the units of the number of base substitutions per site. The rate variation among sites was modelled with a gamma distribution (shape parameter = 1). There were a total of 2,492,438 positions in the final dataset. Evolutionary analyses were conducted in MEGA11. (D) Phylogeny of *Paeniclostridium sordellii* isolates. The tree is based on core genome SNPs determined with snippy 4.6.0. Clustering was inferred with neighbour‐joining. The optimal tree is shown. The percentage of replicate trees in which the associated taxa clustered together in the bootstrap test (100 replicates) is shown above the branches. The tree is drawn to scale, with branch lengths in the same units as those of the evolutionary distances used to infer the phylogenetic tree. The evolutionary distances were computed using the Maximum Composite Likelihood method and are in the units of the number of base substitutions per site. The rate variation among sites was modelled with a gamma distribution (shape parameter = 0.5). There were a total of 2,868,821 positions in the final dataset. Evolutionary analyses were conducted in MEGA11.

**FIGURE 3:**
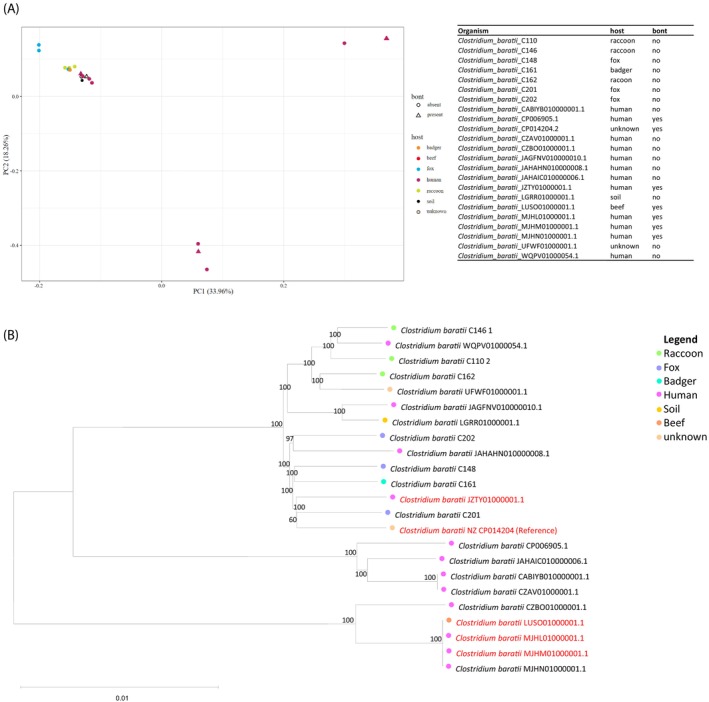
Pan‐genome‐based clustering of *Clostridium baratii*. (A) Pan‐genome‐based principal component analysis based on 23 *C. baratii* genomes. Seven genomes from this study and 16 genomes deposited at GenBank were used. Host and presence of the Botulinum Neurotoxin gene (bont) are indicated by colour code and shapes, respectively. (B) Neighbour‐joining phylogenetic tree based on core genome single nucleotide polymorphisms of 23 *C. baratii* genomes. Seven genomes from this study and 16 genomes deposited at GenBank were used. Genomes encoding botulinum neurotoxin are marked in red. The percentage of replicate trees in which the associated taxa clustered together in the bootstrap test (100 replicates) is shown next to the branches. Evolutionary distances were computed using the Maximum Composite Likelihood method and are in the units of the number of base substitutions per site. The rate variation among sites was modelled with a gamma distribution (shape parameter = 0.5). All positions containing gaps and missing data were eliminated (complete deletion option). There were a total of 2,257,623 positions in the final dataset. Evolutionary analyses were conducted in MEGA11.

We isolated *C. perfringens* from most specimens (Table 1). All strains were type A with 16 out of 58 isolates carrying an additional β2‐toxin‐encoding gene. Besides this, we isolated botulinum toxin‐negative *C. baratii* from seven animals (Figure 2). These strains are closely related to toxin‐carrying isolates from human cases (Figure 3). *Paeniclostridium sordellii* was isolated from six animals. The pathogenic *Listeria monocytogenes* and *Listeria ivanovii* were isolated from five animals and one animal, respectively. Apathogenic species *Listeria seeligeri* and *Listeria innocua* were also isolated (Table 1). *Salmonella* Choleraesuis (ST‐145) was detected in two raccoon dogs, also not native to Germany. Moreover, we isolated different *Yersinia* (*Y*.) species and characterised them for the presence of a type III secretion system‐encoding large plasmid, which was present in one *Y. enterocolitica* strain from a raccoon. This isolate also carried the chromosomal attachment and invasion locus (*ail*). A total of 12 *Y. enterocolitica* were isolated (Table 1, Figure 2). *Yersinia intermedia* was isolated from two raccoons and *Yersinia bercovieri* from one raccoon. Acquired resistance genes were detected in several strains. The *Salmonella* Cholerasuis strain encodes an Aac(6′)‐Iy/Iaa family aminoglycoside 6′‐N‐acetyltransferase conferring resistance to amikacin and tobramycin, as well as a *parC* T57S point mutation leading to ciprofloxacin resistance. *C. baratii* strain C146 harbours a tetracycline efflux MFS transporter TetA(P)‐encoding gene. *P. sordellii* strain C125 encodes the Tet P determinant, which is comprised of two overlapping genes: *tetA*(P) (efflux pump), and *tetB*(P) (ribosomal protection) which have been previously described in *P. sordellii* (Vidor et al., 2019).

**TABLE 1:** Summary of detected bacterial pathogens.

Pathogen species	Host species (no. positive/no. tested)	MLST sequence types (if available)
*Clostridium baratii*	Red fox (3/39), raccoon (3/22), badger (1/8)	NA
*Clostridium colicanis*	Red fox (3/39), badger (1/8)	NA
*Clostridium perfringens*	Red fox (31/39), raccoon (14/22), badger (8/8), raccoon dog (3/3) marten (2/3)	ND
*Paeniclostridium sordellii*	Red fox (4/39), raccoon (1/22), badger (1/8)	NA
*Listeria innocua*	Raccoon dog (1/3)	ST‐637
*Listeria ivanovii*	Raccoon (1/22)	NA
*Listeria monocytogenes*	Red fox (3/39), raccoon (2/22), badger (1/8)	ST‐32, ST‐388 (3x), ST‐883, ST‐3052
*Listeria seeligeri*	Raccoon (2/22)	NA
*Salmonella* Choleraesuis	Raccoon dog (2/3)	ST‐145
*Yersinia bercovieri*	Raccoon (1/22)	NA
*Yersinia enterocolitica*	Red fox (4/39), raccoon (7/22), badger (1/8)	NA
*Yersinia intermedia*	Raccoon (2/22)	NA

Abbreviation: MLST, multi‐locus sequence typing; NA, not available; ND, not done.

## DISCUSSION

Here, we investigated the spatial extent of gut parasite infections and selected bacteria in a range of predators in eastern Germany. We showed that *B. procyonis* spread further northeast than previously reported. A recent representative study investigating samples from 2008 to 2018 did not report it in the state of Brandenburg in northeastern Germany (Heddergott et al., 2020), but highlighted its geographic expansion in Germany. These new data indicate that the parasite is spreading rapidly in the region, and humans in this area must also be considered at risk of getting infected with *B. procyonis*.

With respect to the bacteria detected, *C. baratii* is of special interest, since the natural reservoir of this species has so far not been described (Halpin et al., 2018; Moodley et al., 2015; Silva‐Andrade et al., 2022). While the strains from the current study did not encode botulinum toxin, they were closely related to toxin‐carrying strains based both on the core and pan‐genome (Figure 3). Therefore, other wild predators or even pets may carry *C. baratii*, which requires further investigation. The *P. sordellii* strain harbouring the tetracycline resistance determinant is also of note since tetracyclines are a known treatment option for gas gangrene in humans (Vidor et al., 2019). However, none of the *P. sordellii* isolates from the current study encoded the lethal toxin conferring increased virulence (Bernard et al., 2022). While we isolated *Y. enterocolitica* from 12 out of 77 samples, only one strain isolated from a raccoon harboured the known virulence plasmid and can thus be considered pathogenic. *Salmonella* Choleraesuis ST‐145 is mainly found in wild boar but has also been reported in foxes in Germany by Uelze et al. (2021). Interestingly, Uelze et al. also reported that aminoglycoside resistance genes and ciprofloxacin resistance conferring mutations, which were however, different from the gene and mutation detected in our strain.

Phylogenetically, the strains of individual species were polyclonal, not pointing towards specific wildlife‐associated strains (Figure 2). *Salmonella* Choleraesuis, of which we isolated two strains, may be an exception since we also found ST‐145, which is the major type found in German wild animals (Uelze et al., 2021).

In conclusion, we detected *C. baratii* among other *Clostridia* spp. for the first time in wild predators. Even though none of the strains harboured the botulinum toxin, they were phylogenetically closely related to toxigenic strains. Further studies are necessary to elucidate if badgers, foxes or raccoons are potential reservoir hosts for toxigenic strains. Besides this, we detected other zoonotic bacteria, such as *Y. enterocolitica* or *L. monocytogenes*. We detected a narrow spectrum of antibiotic resistance genes, which do not point towards the reservoir role of the predators. Overall, our data indicate a low risk for humans with respect to exposure to predator‐derived pathogens investigated here except the raccoon, an invasive species in the region concerned. Here, the further spread of the parasite *B. procyonis* is of concern as it poses a zoonotic risk with the possibility of severe or even fatal disease in the human host.

## AUTHOR CONTRIBUTIONS

**Sonja Kittl:** Conceptualization (equal); data curation (equal); formal analysis (equal); visualization (lead); writing – original draft (equal); writing – review and editing (equal). **Caroline F. Frey:** Investigation (equal); methodology (equal); writing – review and editing (equal). **Isabelle Brodard:** Investigation (equal); methodology (equal); writing – review and editing (equal). **Nadia Scalisi:** Data curation (equal); investigation (equal). **Maria Elena Vargas Amado:** Visualization (equal); writing – review and editing (equal). **Andreas Thomann:** Investigation (equal); methodology (equal). **Peter Schierack:** Conceptualization (equal); writing – review and editing (equal). **Joerg Jores:** Conceptualization (lead); data curation (equal); funding acquisition (lead); methodology (equal); project administration (lead); supervision (lead); visualization (equal); writing – original draft (equal); writing – review and editing (equal).

## CONFLICT OF INTEREST STATEMENT

The authors declare no conflicts of interest.

## Supporting information


**Data S1.** Supporting Information.

## Data Availability

The sequencing data is available from GenBank (PRJNA961371).
